# Supplementary material for: Genome-wide association meta-analysis yields 20 loci associated with gallstone disease
Source: Nat Commun. 2018 Nov 30;9:5101. doi: 10.1038/s41467-018-07460-y (PMC6269469; doi:10.1038/s41467-018-07460-y)
Supplement: Supplementary file 1 — Supplementary Information [file 41467_2018_7460_MOESM1_ESM.pdf]

Supplementary information for

Genome-wide association meta-analysis yields 20 loci associated with gallstone disease

Ferkingstad et al.

Supplementary Tables

Supplementary Table 1. *Demographic information for gallstone cases and population controls in Iceland and the UK used in the current study. BMI = body mass index, T2D = type 2 diabetes*

	Iceland		UK	
	**Cases**	**Controls**	**Cases**	**Controls**
N	8,785	348,356	18,417	390,241
Males/Females	2,727/6,058	178,473/169,883	50,51/13,366	182,668/207,570
Age (sd)	69 (18)	48 (24)	60.0 (7.4)	57.8 (8.0)
BMI (sd)	28.76 (5.80), N = 4,597	25.17 (5.95), N = 84,646	29.8 (5.5), N = 18,333	27.3 (4.7), N = 389,031
T2D	1,006 (11.5%)	10,347 (3.0%)	1,856 (10.1%)	20,533 (5.26%)

Supplementary Table 2. *Variants that represent known gallstone disease signals in the present meta-analysis of Icelandic and UK Biobank data. For Iceland (N_{cases} = 8,757 and $N_{controls}$ = 346,688), UK BioBank (N_{cases} = 18,417 and $N_{controls}$ = 390,150), in total N_{cases}= 27,174 and $N_{controls}$ = 736,838). Effect is shown for the minor allele. MAF: Minor allele frequency in Iceland, min: Minor allele, maj: Major allele, OR: Odds ratio, CI: Confidence interval, PMID = PubMed ID of original report.*

**Marker**	**Position (Hg38)**	**minA/majA**	**$MAF_{Iceland}$ (%)**	**Consequence**	**Gene**	**OR**	**P**	**PMID**
rs11887534	chr2:43839108	C/G	5.5	missense	*ABCG8*	1.94 [1.88,2.08]	1.0×10^{-353}	27094239, 17632509
rs212100	chr19:47873738	T/C	16	intron	*SULT2A1*	0.86 [0.83,0.88]	2.0×10^{-31}	27094239
rs12633863	chr3:149493725	G/A	45	intron	*TM4SF4*	1.11 [1.09,1.13]	3.6×10^{-30}	27094239
rs4148808	chr7:87476479	C/T	16	upstream	*ABCB4*	0.86 [0.84,0.88]	8.6×10^{-28}	25807286
rs6471717	chr8:58464798	G/A	31	intergenic	*CYP7A1*	1.11 [1.09,1.13]	9.9×10^{-26}	27094239
rs686030	chr9:15304784	C/A	13	intron	*TTC39B*	0.88 [0.85,0.90]	1.8×10^{-20}	27094239
rs756082276	chr7:87443343	CCT/C	0.14	frameshift	*ABCB4*	4.13 [2.98,5.72]	1.2×10^{-17}	25807286
rs1260326	chr2:27508073	T/C	34	missense	*GCKR*	0.93 [0.91,0.94]	2.0×10^{-16}	27094239
rs756935975	chr7:87431432	T/C	0.20	missense	*ABCB4*	3.09 [2.32,4.12]	1.8×10^{-14}	25807286
rs2070959	chr2:233693545	G/A	32	missense	*UGT1A6*	1.07 [1.05,1.09]	8.5×10^{-11}	26039129
rs45575636	chr7:87431528	T/C	0.31	missense	*ABCB4*	1.34 [1.22,1.48]	7.9×10^{-9}	25807286

Supplementary Table 3. *Significant eQTLs among the reported novel gallstone associated variants. Top eQTL variant: most significant eQTL in the region. r^2: r^2 between reported marker and top eQTL marker. Effects are for the minor allele and in standard deviation units. Directions of effects: Whether effects on gallstone disease risk and gene expression are in the same direction (i.e. the allele that increases gallstone disease risk also increases gene expression) or in opposite directions.*

Reported SNPID[minA]	Top eQTL SNPID[minA]	r^2	Tissue	eQTL gene	Reported marker		Top eQTL marker		Data source	Directions of effects on gallstone risk and gene expression
					Effect	P-value	Effect	P-value		
rs601338[G]	rs601338[G]	1.00	Esophagus – Mucosa*	FUT2	-0.72	1.4×10^{-102}	-0.72	1.4×10^{-102}	GTEx (N=358)	Same
rs2469991[T]	rs2470043[A]	0.82	Adipose - Subcutaneous	MAL2	-0.62	3.8×10^{-33}	-0.65	8.7×10^{-41}	GTEx (N=385)	Same
rs11641445[T]	rs11644920[T]	0.97	Adipose - Subcutaneous	LITAF	-0.49	4.2×10^{-35}	-0.52	1.5×10^{-41}	GTEx (N=385)	Opposite
rs11641445[T]	rs11644920[T]	0.97	Liver	LITAF	-0.30	8.4×10^{-7}	-0.34	3.2×10^{-8}	GTEx (N=153)	Opposite

*Tissue with the most significant eQTL, also reported for other tissues in GTEx.

Supplementary Table 4. *Effects of the SLC10A2 (NP_000443.1) missense variants Pro290Ser and Val98Ile, unadjusted and adjusted for Val98Ile and Pro290Ser, respectively, in the combined Icelandic and UK data (N_{cases}= 27,174 and $N_{controls}$ = 736,838).*

		**Unadjusted**		**Adjusted for other marker**	
**SNPID**	**HGVS.p**	**OR (95% CI)**	**P-value**	**OR (95% CI)**	**P-value**
rs56398830	Pro290Ser	1.36 (1.25, 1.49)	2.1×10^{-12}	1.42 (1.29, 1.56)	2.1×10^{-13}
rs55971546	Val98Ile	1.15 (1.10, 1.20)	1.8×10^{-10}	1.17 (1.11, 1.22)	2.5×10^{-11}

Supplementary Table 5. *Levels of serum taurocholate (N = 273) and fibroblast growth factor 19 (FGF19, N = 293) for four carriers of Pro290Ser (all heterozygous). Measurements are centered to have mean zero and standard deviation one .*

**Pro290Ser carrier**	**Taurocholate**	**Fibroblast growth factor 19 (FGF19)**
1	-1.67	-0.61
2	-1.33	1.12
3	-1.71	-1.10
4	-1.51	0.25

Supplementary Table 6. *Missense variants in SLC10A2 (NP_000443.1) tested in the gallstone disease meta-analysis of Icelandic and UK BioBank data. Significance levels and effects are shown separately for the Icelandic and UK datasets and for the combined analysis. MAF: Minor allele frequency, min: Minor allele, maj: Major allele, OR: Odds ratio, CI: Confidence interval,* P_{het}*: heterogeneity P-value.*

			**Iceland cases = 8,757 / ctrl = 346,688**			**UKBB cases = 18,417 / ctrl = 390,150**			**Combined cases= 27,174 / ctrl = 736,838**		
**HGVS.p**	**Position**	**min/ maj**	**OR (95% CI)**	**P-value**	**MAF (%)**	**OR (95% CI)**	**P-value**	**MAF (%)**	**OR (95% CI)**	**P-value**	P_{het}
Pro290Ser	chr13:103049340	A/G	1.58 (1.28, 1.96)	2.3 × 10-5	0.59	1.32 (1.20, 1.46)	6.3×10^{-9}	1.1	1.36 (1.25, 1.49)	2.1×10^{-12}	0.14
Val98Ile	chr13:103065958	T/C	1.15 (1.06, 1.26)	0.0015	4.1	1.15 (1.10, 1.21)	3.3×10^{-8}	4.1	1.15 (1.10, 1.20)	1.8×10^{-10}	0.97
Pro65Leu	chr13:103066056	A/G	1.54 (1.15, 2.06)	0.0042	0.26	1.30 (0.98, 1.71)	0.066	0.14	1.40 (1.15, 1.72)	0.00096	0.41
Pro142Leu	chr13:103058335	A/G	0.02 (0.00, 17.26)	0.25	0.005	1.52 (1.08, 2.14)	0.016	0.078	1.50 (1.07, 2.11)	0.019	0.2
Thr167Ile	chr13:103052705	A/G	0.45 (0.13, 1.60)	0.22	0.032	0.72 (0.45, 1.16)	0.18	0.066	0.68 (0.44, 1.06)	0.09	0.5
Asn27Ser	chr13:103066170	C/T	1.93 (0.92, 4.03)	0.082	0.045	0.96 (0.21, 4.35)	0.96	0.023	1.63 (0.86, 3.11)	0.14	0.37
Phe304Leu	chr13:103049298	G/A	0.87 (0.63, 1.21)	0.41	0.38	0.90 (0.77, 1.06)	0.21	0.44	0.90 (0.78, 1.04)	0.14	0.85
Trp66Ter	chr13:103066053	T/C	-	-	0	1.48 (0.85, 2.56)	0.16	0.03	-	-	-
Gly77Glu	chr13:103066020	T/C	-	-	0.008	1.98 (0.55, 7.09)	0.29	0.008	-	-	-
Ala202Ser	chr13:103051414	A/C	-	-	0	2.75 (0.24, 31.91)	0.42	0.003	-	-	-
Leu215Ser	chr13:103051374	G/A	1.77 (0.43, 7.27)	0.43	0.01	-	-	0	-	-	-
Ala219Thr	chr13:103051363	T/C	-	-	0	2.31 (0.22, 24.55)	0.49	0.004	-	-	-
Ser171Ala	chr13:103052694	C/A	0.93 (0.88, 0.98)	0.0097	11	1.01 (0.98, 1.05)	0.49	12	0.99 (0.96, 1.02)	0.49	0.01
Val159Ile	chr13:103058285	T/C	1.07 (0.92, 1.24)	0.37	1.4	1.01 (0.93, 1.09)	0.84	1.9	1.02 (0.95, 1.09)	0.55	0.49
Arg256Ter	chr13:103049442	A/G	-	-	0	1.15 (0.49, 2.71)	0.75	0.014	-	-	-
Val100Ala	chr13:103065951	G/A	-	-	0	1.19 (0.37, 3.86)	0.77	0.011	-	-	-
Asp154Asn	chr13:103058300	T/C	0.71 (0.33, 1.51)	0.37	0.11	1.43 (0.57, 3.61)	0.45	0.02	0.94 (0.52, 1.68)	0.83	0.25
Phe296Leu	chr13:103049322	G/A	0.89 (0.60, 1.30)	0.54	0.25	1.04 (0.87, 1.23)	0.68	0.48	1.01 (0.86, 1.18)	0.9	0.46
Gly77Val	chr13:103066020	A/C	1.09 (0.14, 8.27)	0.94	0.008			0.008	1.09 (0.14, 8.27)	0.94	-

Supplementary Table 7. *Quantitative traits associated with SERPINA Pi Z allele (rs28929474; Glu366Lys) in the Icelandic population. β denotes the effect measured in standard deviations.*

Trait	N	β (95% CI)	P-value
Alpha-1-antitrypsin	6,452	-1.71 (-1.88, -1.53)	1.0×10^{-83}
Alpha-fetoprotein	19,125	-0.84 (-0.95, -0.72)	5.5×10^{-47}
Alkaline phosphatase	154,097	0.25 (0.20, 0.31)	2.7×10^{-20}
Alanine transaminase	172,086	0.18 (0.14, 0.22)	2.7×10^{-20}
Gamma glutamyl transpeptidase	156,692	0.12 (0.08, 0.17)	4.2×10^{-9}
Platelet count	268,487	0.09 (0.06, 0.12)	8.1×10^{-8}

Supplementary Table 8. *ICD-10 and OPCS procedure codes indicative of gallstone disease assigned to UK PI ZZ carriers. YOB = year of birth, AGE = Age at gallstone disease diagnosis.*

PI ZZ carriers	SEX	YOB	AGE	ICD code	OPCS code
I	Male	1961	44	(K80.1) Calculus of gallbladder with other cholecystitis	(J18.3) Total Cholecystectomy
II	Female	1959	44	(K80.2) Calculus of gallbladder without cholecystitis	(J18.3) Total Cholecystectomy
III	Female	1947	66	(K80.0) Calculus of gallbladder with acute cholecystitis	(J18.3) Total Cholecystectomy
IV	Male	1963	48	(K80.0) Calculus of gallbladder with acute cholecystitis	(J18.5) Partial Cholecystectomy
V	Female	1950	55	(K80.1) Calculus of gallbladder with other cholecystitis	(J18.3) Total Cholecystectomy
VI	Female	1940	60	(K80.2) Calculus of gallbladder without cholecystitis	(J18.3) Total Cholecystectomy
VII	Female	1960		(K80.2) Calculus of gallbladder without cholecystitis	(J18.3) Total Cholecystectomy
VIII	Female	1947	66	(K80.2) Calculus of gallbladder without cholecystitis	
IX	Male	1946	68	(K80.0) Calculus of gallbladder with acute cholecystitis	(J18.3) Total Cholecystectomy

Supplementary Table 9. *Quantitative traits associated with the HNF4A Thr139Ile variant. Data from a recent exome-wide associate study of plasma lipids (PMID 29083408; data available on-line from the Global Lipids Genetics Consortium (GLGC), http://csg.sph.umich.edu/abecasis/public/lipids2017/) and from Iceland. β denotes the effect measured in standard deviations.*

Trait	N	β (95% CI)	P-value
LDL cholesterol (GLGC)	>300,000	-0.05 (-0.06,-0.04)	8.4×10^{-13}
HDL cholesterol (GLGC)	>300,000	-0.14 (-0.15, -0.13)	1.6×10^{-81}
HDL cholesterol (Iceland)	136,736	-0.15 (-0.18, -0.13)	2.4×10^{-33}
Total cholesterol (GLGC)	>300,000	-0.09 (-0.11,-0.07)	5.5×10^{-14}
Total cholesterol (Iceland)	150,211	-0.10 (-0.12, -0.07)	6.5×10^{-16}
Hemoglobin (Iceland)	273,160	0.03 (0.02, 0.05)	1.2×10^{-7}
Bilirubin (Iceland)	109,748	0.04 (0.02, 0.06)	1.3×10^{-5}
Gamma glutamyl transpeptidase (Iceland)	156,692	0.04 (0.02, 0.05)	2.8×10^{-5}

Supplementary Table 10**:** *Case-control (cc) and quantitative (qt) phenotypes associating with FUT2 wild-type secretor allele rs601338[G]. Sample sizes for case-control phenotypes are listed as N_{cases} / $N_{controls}$. Effects are in units of standard deviations.*

**Effect**	**P-value**	**Trait type**	**Phenotype**	**Sample size**
1.56 (1.34, 1.82)	7.20×10^{-9}	cc	Viral and other intestinal infections (ICD-10 A08*)	439 / 357,766
1.97 (1.54, 2.50)	4.28×10^{-8}	cc	Acute gastroenteropathy due to Norwalk agent (ICD-10 A08.1)	159 / 327,399
-0.18 (-0.19, -0.17)	2.80×10^{-300}	qt	B12	157,081
-0.46 (-0.49, -0.44)	5.57×10^{-300}	qt	CA 19-9	10,560
0.11 (0.10, 0.12)	7.62×10^{-156}	qt	ALP	170,276
-0.23 (-0.25, -0.21)	2.16×10^{-150}	qt	CEA	23,080
-0.11 (-0.12, -0.10)	7.89×10^{-81}	qt	Lipase	54,395
-0.04 (-0.05, -0.03)	2.34×10^{-16}	qt	Amylase	75,177
0.03 (0.02, 0.03)	2.73×10^{-15}	qt	Ferritin	172,918
-0.04 (-0.05, -0.03)	8.89×10^{-15}	qt	Total cholesterol	151,050
-0.04 (-0.05, -0.03)	2.12×10^{-13}	qt	LDL cholesterol	126,873
-0.03 (-0.04, -0.02)	7.09×10^{-12}	qt	Non-HDL cholesterol	137,052
-0.02 (-0.03, -0.02)	1.03×10^{-09}	qt	GGT	172,304
0.03 (0.02, 0.04)	1.78×10^{-08}	qt	Uric acid	76,515
0.41 (0.26, 0.56)	7.91×10^{-08}	qt	FAM3D (Proteomics)	294
-0.09 (-0.13, -0.06)	1.71×10^{-07}	qt	CA 125	7,696
0.02 (0.01, 0.03)	1.76×10^{-07}	qt	Folate	106,687

Supplementary Table 11: *The two variants with a significant difference of effects between ICD10 code based and self-reported gallstone cases.* P_{het}*: P-value for test of heterogeneity.*

**Variant**	**ICD10 (N = 14,350)**	**Self-reported (N = 4,067)**	**Overall (N = 18,417)**	P_{het}
SERPINA1 Glu366Lys	OR = 1.25 (1.15-1.35); P = 7.0×10^{-8}	OR = 1.67 (1.46-1.91); P = 6.2×10^{-14}	OR = 1.35 (1.26-1.45); P = 5.9×10^{-17}	2.2×10^{-4}
ABCG8 Asp19His	OR = 1.86 (1.78-1.93); P = 2.5×10^{-189}	OR = 2.40 (2.23-2.58); P = 8.7×10^{-125}	OR = 2.00 (1.93-2.07); P = 6.1×10^{-300}	1.6×10^{-9}

Supplementary Table 12. *Gallstone disease association results for rs9514089[C] SLC10A2 intron variant from papers by Renner et al. (2009) (PMID: 19823678), Tönjes et al (2011) (PMID: 22093174) and the Icelandic and UK data of the present paper. The first row shows results for a meta-analysis of the Stuttgard and Aachen cohorts, as reported in Table 2 on p. 4 of Renner et al. (2009). The second row shows results for a follow-up study in the Sorbs cohort, as reported in Table 2 on p. 3 of Tönjes et al (2011). The third, fourth and fifth rows show results from the Icelandic, UK, and Iceland + UK meta-analysis of the present paper. The last row shows results from a meta-analysis of our Icelandic + UK combined data with the combined German data (Stuttgart + Aachen + Sorbs). We note that the latter combined results show no association of rs9514089[C] with gallstone disease, and that the confidence interval from the combined Icelandic + UK + German meta-analysis excludes the confidence interval from the initial study based on the Stuttgar and Aachen cohorts. Thus, we conclude that the initial result from Renner et al. (2009) is not replicated. All results shown are based on the additive genetic model.*

**Analysis (cohort)**	**Authors**	**PMID**	**Year**	**N (cases/controls)**	**MAF (%)**	**P-value**	**OR (95% CI)**
Germany, initial study (Stuttgart + Aachen)	Renner et al.	19823678	2009	240 / 255	36.6	0.0077	2.04 (1.19-3.55)
Germany, follow-up study (Sorbs)	Tönjes et al.	22093174	2011	183 / 826	36.6	0.19	0.83 (0.63-1.09)
Iceland	Current paper	-	2018	8,757 / 346,688	36.4	0.81	1.005 (0.966-1.046)
UK	Current paper	-	2018	18,417 / 390,150	37.3	0.24	0.987 (0.966-1.009)
Iceland + UK meta-analysis	Current paper	-	2018	27,174 / 736,838	36.9	0.36	0.991 (0.973-1.010)
Iceland + UK + Germany meta-analysis	Current paper	-	2018	27,597 / 737,919	36.9	0.80	0.991 (0.973-1.011)

Supplementary Figures

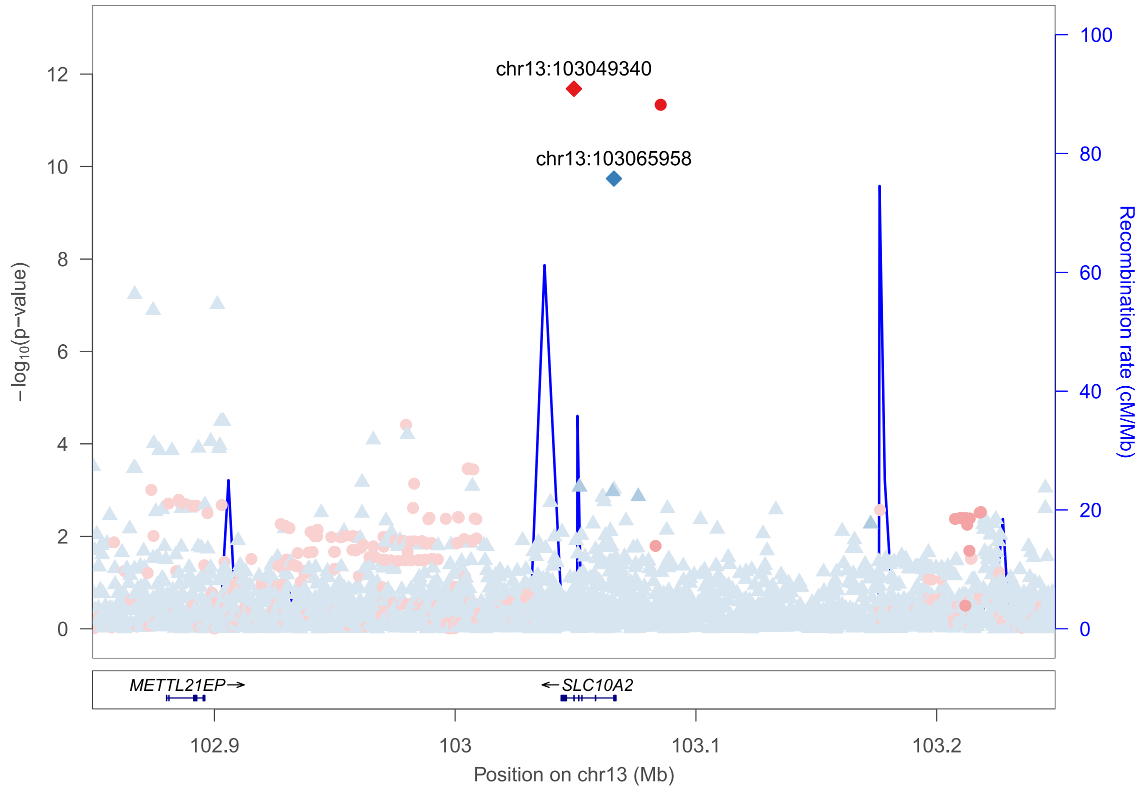

Supplementary Fig. 1. *Locus plot depicting variants at the SLC10A2 locus associating with gallstone disease. The leading variants, NP_000443.1:p.Pro290Ser and NP_000443.1:p.Val98Ile are labelled as diamonds and shown in red and blue, respectively. Other variants are colored according to correlation (r^2) with their leading marker (darker colors indicating stronger correlation). $-log_{10}$ P-values are shown along the left y-axis and correspond to the variants depicted in the plot. The right y-axis shows calculated recombination rates at the chromosomal location, plotted as a solid blue line. Genes are marked below by horizontal blue lines, arrows on the horizontal blue lines show the direction of transcription, and rectangles are exons.*

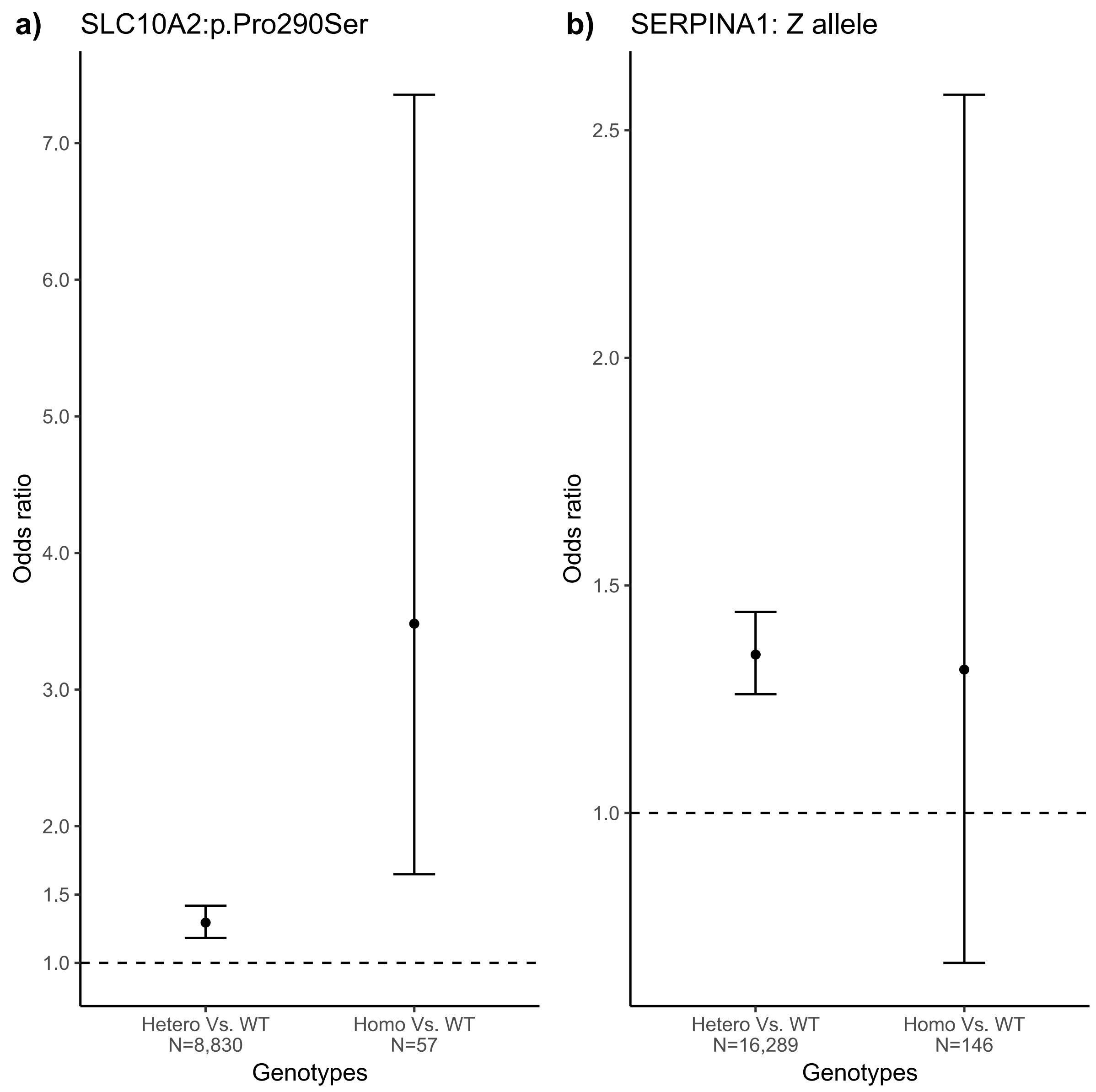

Supplementary Fig. 2. *The effects of SLC10A2:p.Pro290Ser and the AAT/SERPINA1 PI Z genotype classes on gallstone disease. The x-axis in each plot show the heterozygote and homozygous for the minor allele. The y-axis shows gallstone disease odds ratios (with homozygotes for the major allele as reference). The number of carriers behind the computations of each odds ratio is shown on the x-axis below each genotype.*

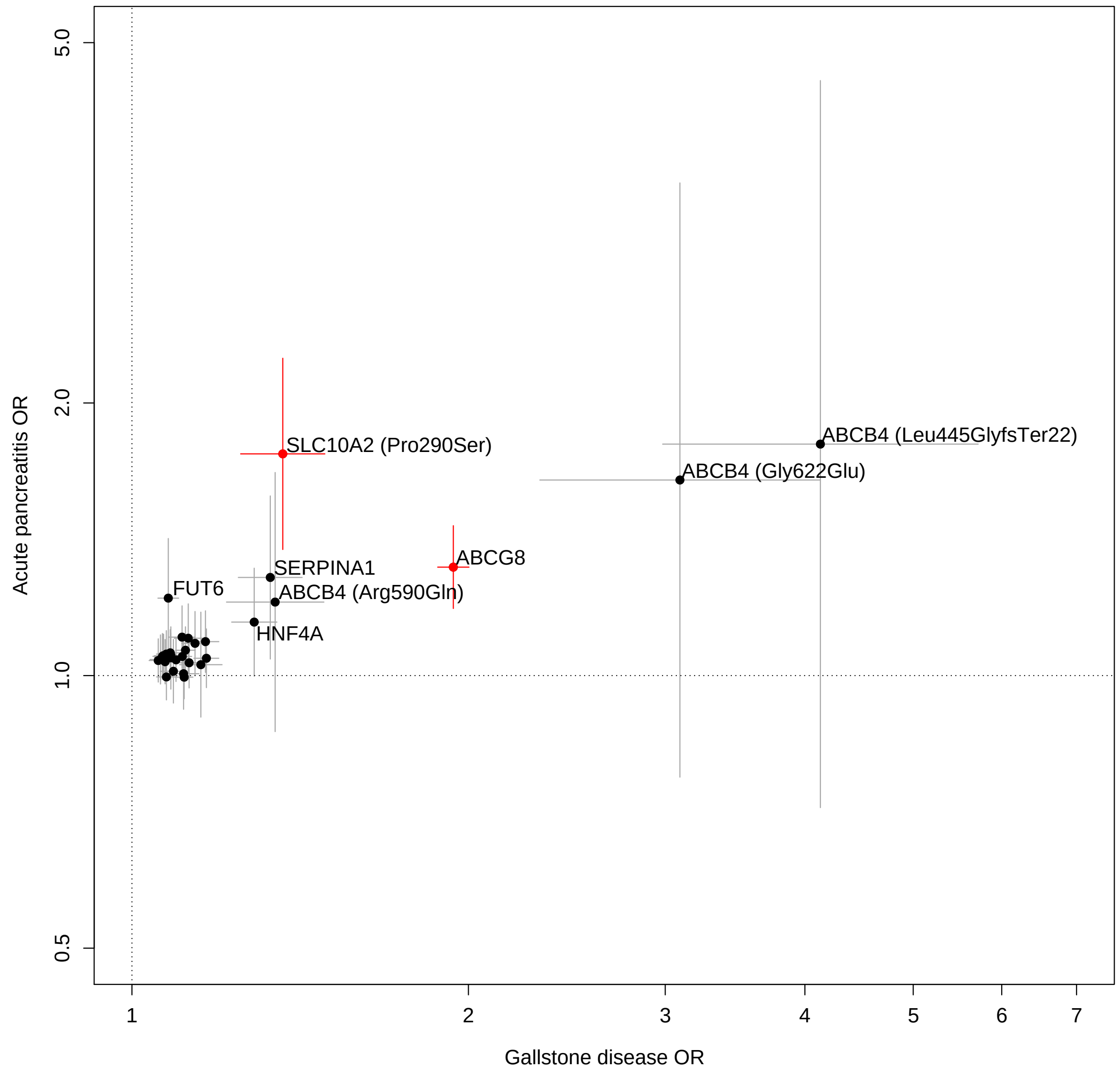

Supplementary Fig. 3. *Scatter plot showing how the 32 variants reported to associate with gallstone disease in the current meta-analysis affect acute pancreatitis risk. The x-axis shows their effect (OR) on gallstone disease in the combined Icelandic and UK biobank dataset (N_{cases}= 27,174 and $N_{controls}$ = 736,838) and the y-axis shows their effect (OR) on acute pancreatitis in the combined Icelandic and UK BioBank dataset (N_{cases}= 2,843 and $N_{controls}$ = 768,171). Variants associated with the highest pancreatitis risk are labeled. Error bars represent 95% confidence intervals. The colors indicate significant P-values for pancreatitis (red: $P < 0.05/32 = 0.0016$, black: $P > 0.0016$).*

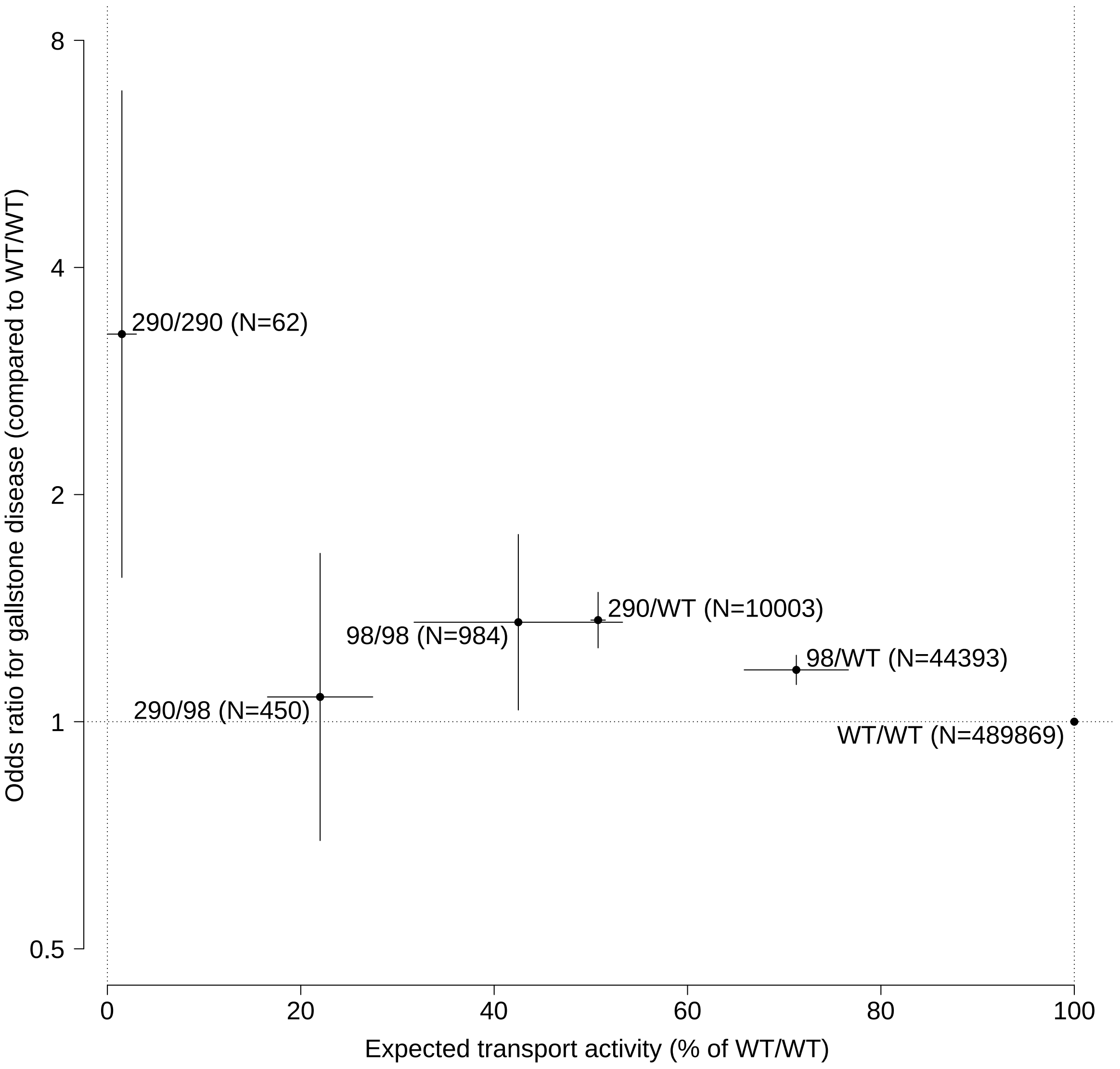

Supplementary Fig. 4. *A scatter plot showing six genotype combinations of Pro290Ser (denoted 290), Val98Ile (denoted 98), and wild-type (denoted WT). The x-axis shows the predicted transport activity of each genotype (total* N_{cases}*= 27,174 and* $N_{controls}$ *= 736,838) and the y-axis shows their effect on gallstone disease risk in the combined Icelandic and UK BioBank dataset (*N_{cases}*=10,857;* $N_{controls}$*=135,512). Numbers in parentheses represent the number carriers of each genotype.*

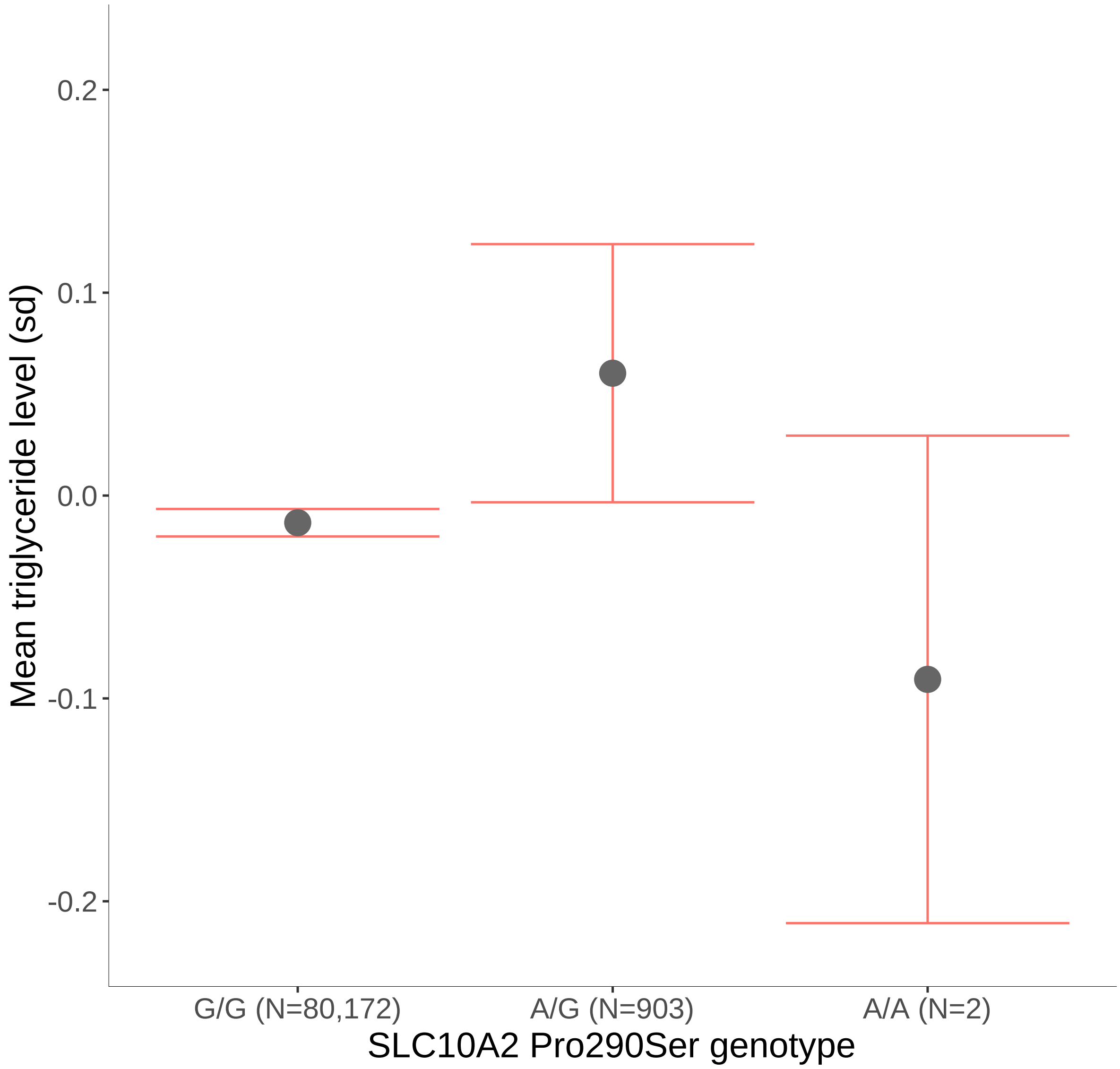

Supplementary Fig. 5. *The effects of SLC10A2 rs56398830 (Pro290Ser) genotype classes on triglyceride levels in the Icelandic data. The x-axis shows the possible genotypes for rs56398830. The y-axis shows mean triglyceride levels, where the units are standard deviations from the overall mean. Errors bars indicate 95% confidence intervals. Numbers in parantheses indicate sample sizes for each genotype class.*

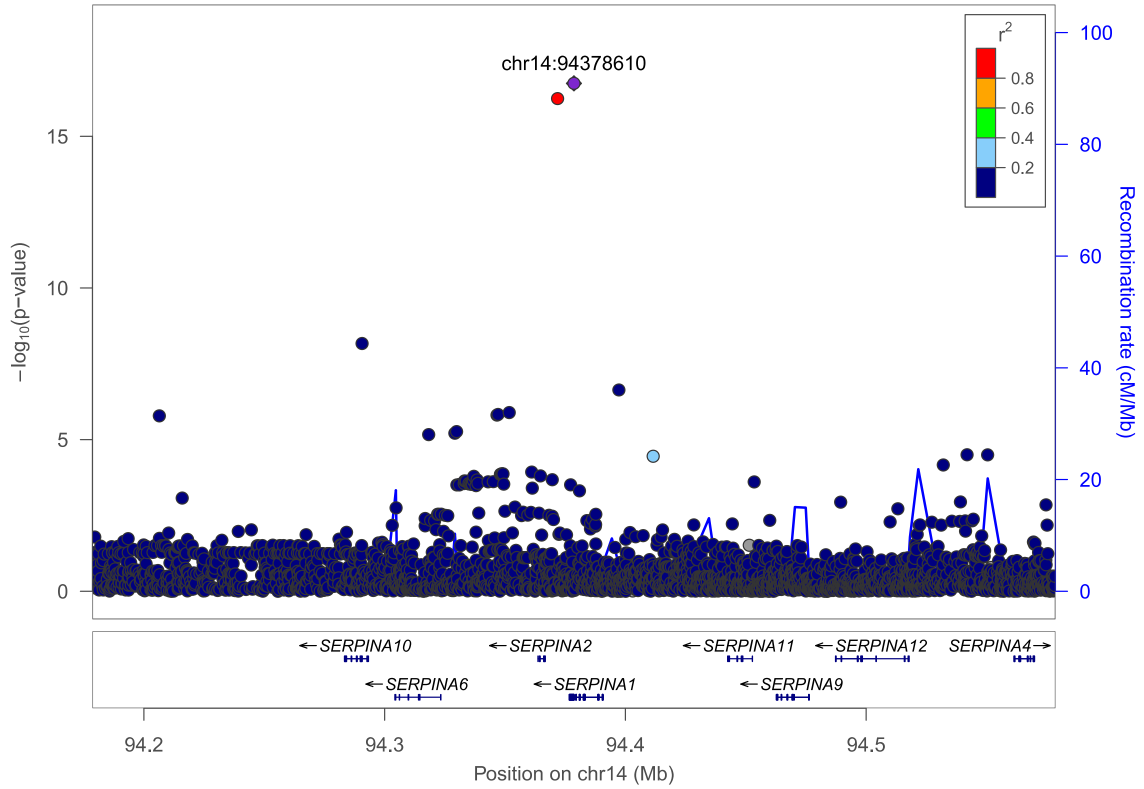

Supplementary Fig. 6. *Locus plot depicting variants at the AAT/SERPINA1 locus associating with gallstone disease. The leading variant PI Z (NP_000286.3:p.Glu366Lys) is labeled as a purple diamond, other variants are colored according to correlation (r^2) with the leading marker (legend at top-right). LD data are based on the Icelandic dataset. P-values are from the gallstone disease meta-analysis of the combined Icelandic and UK BioBank datasets. -log_{10} P-values are shown along the left y-axis and correspond to the variants depicted in the plot. The right y-axis shows calculated recombination rates at the chromosomal location, plotted as a solid blue line. Genes are marked below by horizontal blue lines, arrows on the horizontal blue lines show the direction of transcription, and rectangles are exons.*

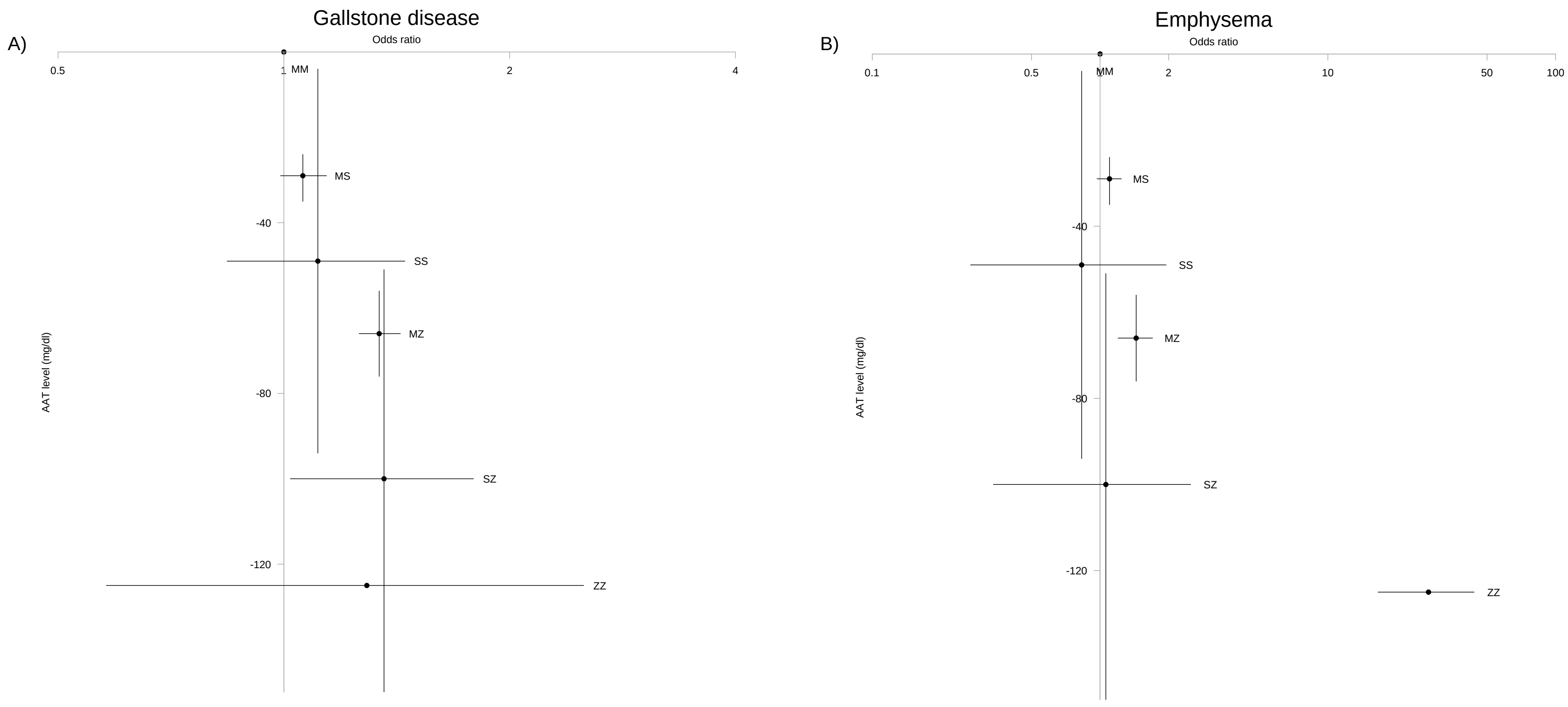

Supplementary Fig. 7. *Two scatter plots showing classical AAT (SERPINA1) genotypes*. The y-axes show their effect on serum AAT levels in Icelandic dataset (total N = 6,452). The x-axes shows their effect on A) gallstone disease risk in the combined Icelandic and UK BioBank dataset (N_{cases}= 27,174 and $N_{controls}$ = 736,838), and B) emphysema risk in the combined Icelandic and UK BioBank dataset (N_{cases}= 3,252 and $N_{controls}$ = 596,760).*

**The effect of PI ZZ on serum AAT levels was estimated from a study by Donato et al. (PMID: 22912357) as no PI ZZ carriers had AAT measurements in the Icelandic dataset.*

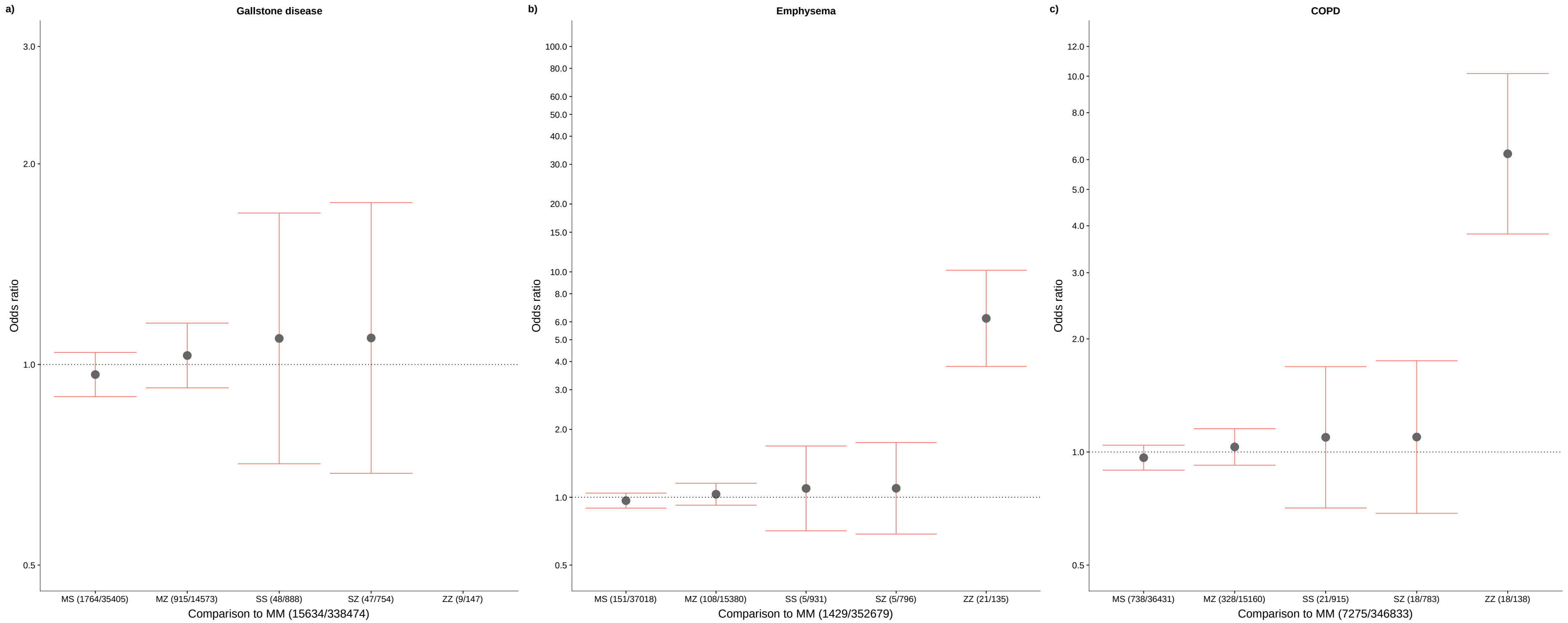

Supplementary Fig. 8. *Genotypic risk for classical AAT/SERPINA1 alleles with gallstone disease, emphysema and COPD. The x-axis in each plot show the classical AAT/SERPINA1 alleles. The y-axis shows gallstone disease logarithmic odds ratios (with the MM allele as reference). The number of cases behind the computations of each odds ratio is shown on the x-axis below each genotype.*

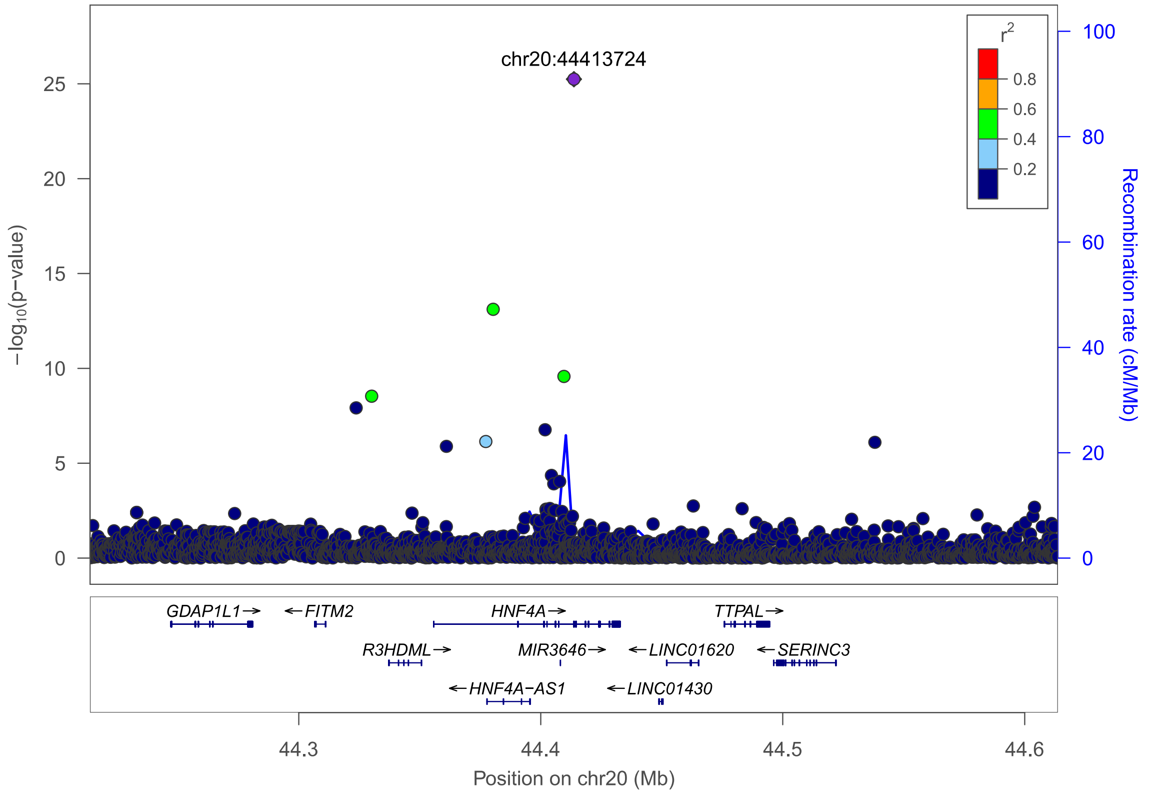

Supplementary Fig. 9. *Locus plot depicting variants at the HNF4A locus associating with gallstone disease. The leading variant rs1800961/Thr139Ile in HNF4A (NP_000448.3) is labeled as a purple diamond, other variants are colored according to correlation (r^2) with the leading marker (legend at top-right). LD data are based on the Icelandic dataset. P-values are from the gallstone disease meta-analysis of the combined Icelandic and UK BioBank datasets. -log_{10} P-values are shown along the left y-axis and correspond to the variants depicted in the plot. The right y-axis shows calculated recombination rates at the chromosomal location, plotted as a solid blue line. Genes are marked below by horizontal blue lines, arrows on the horizontal blue lines show the direction of transcription, and rectangles are exons.*

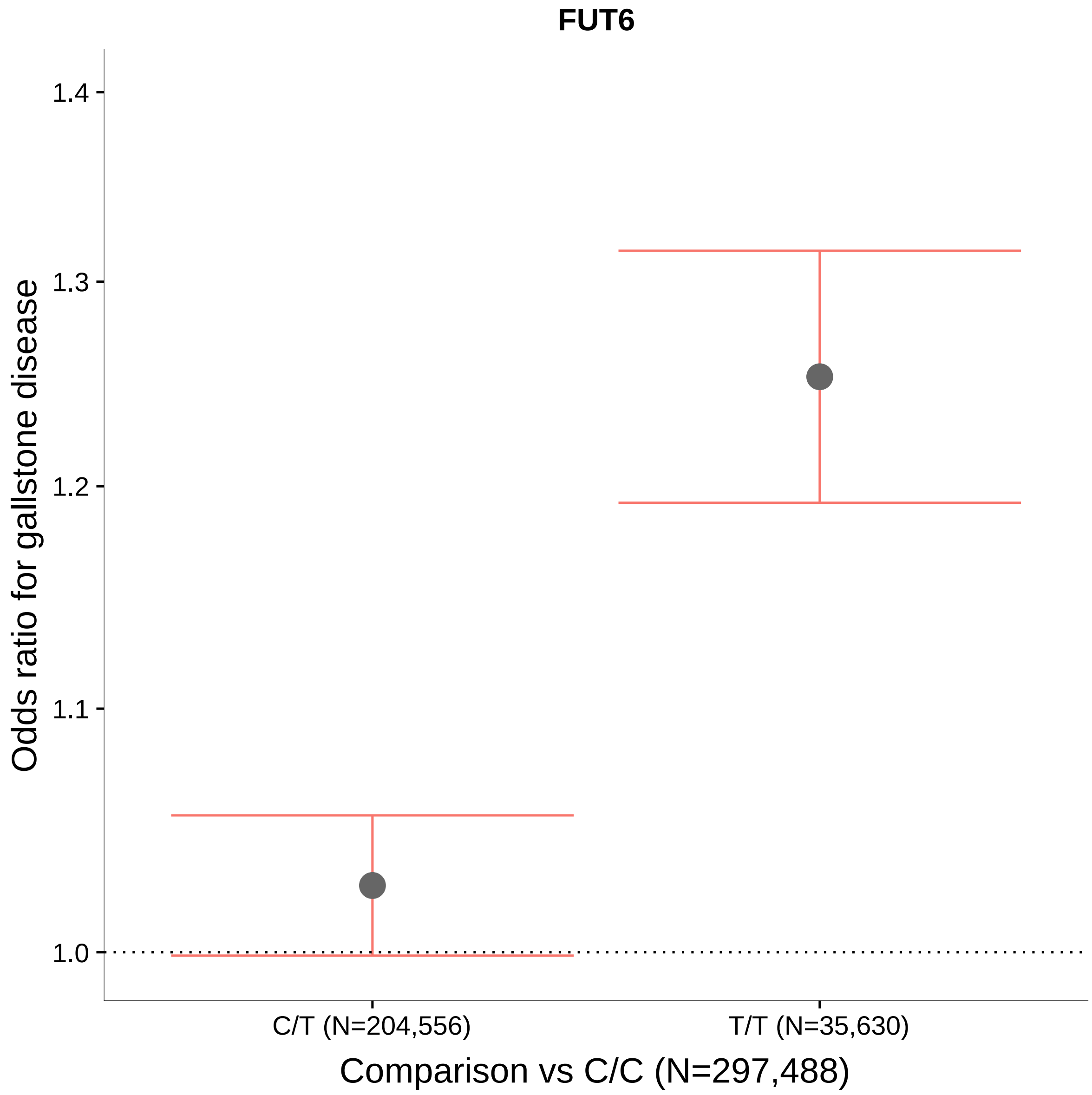

Supplementary Fig. 10: *The effects of FUT6 rs708686[T] genotype classes on gallstone disease in the combined Icelandic and UK data. The x-axis shows the heterozygote and homozygous for the minor allele rs708686[T]. The y-axis shows gallstone disease odds ratios for heterozygous and homozygous FUT6 rs708686[T] carriers (with homozygous major allele carriers as reference). Errors bars indicate 95% confidence intervals.*

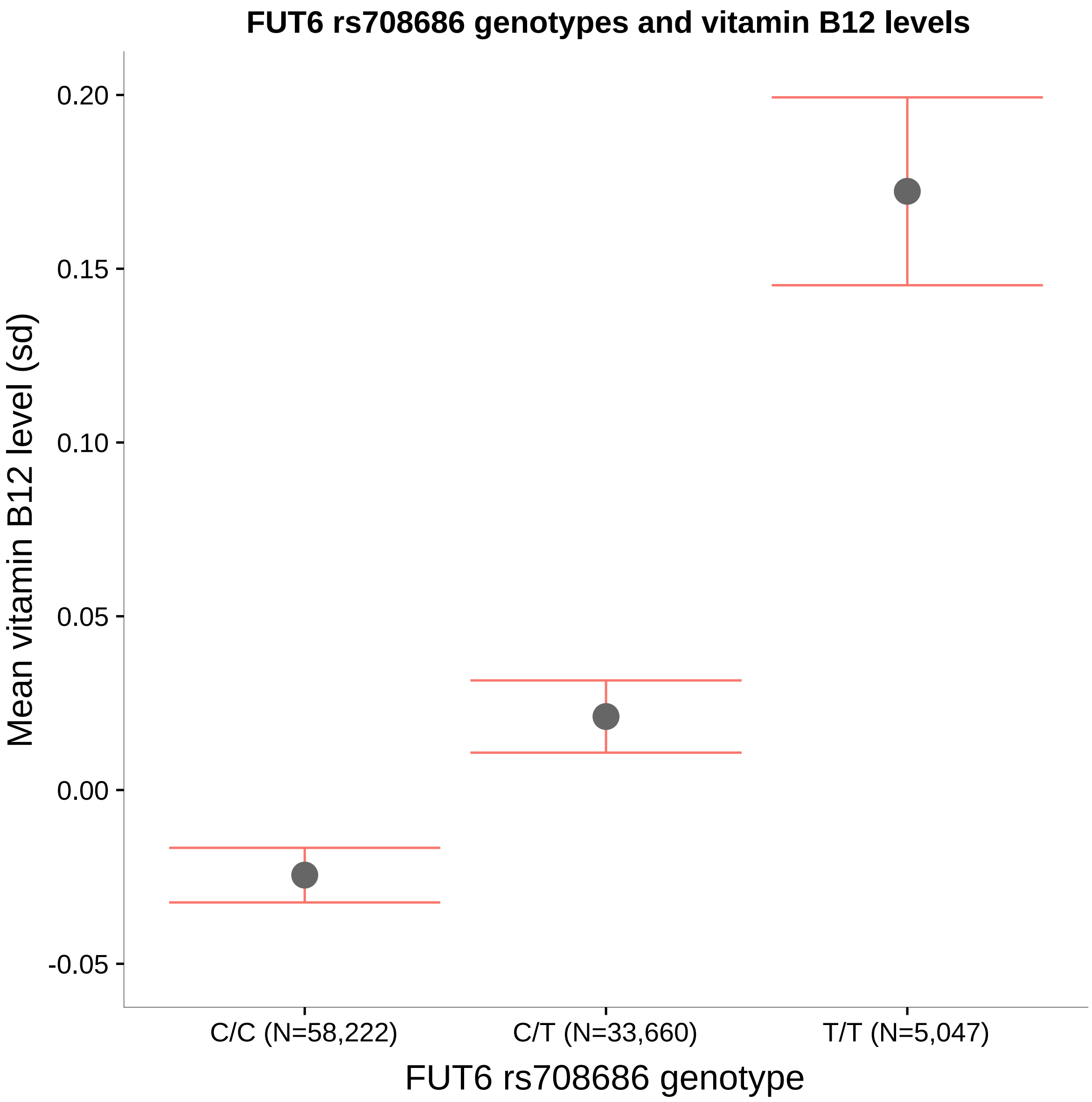

Supplementary Fig. 11. *The effects of FUT6 rs708686 genotype classes on vitamin B12 levels in the Icelandic data. The x-axis shows the possible genotypes for rs708686. The y-axis shows mean vitamin B12 levels, where the units are standard deviations from the overall mean. Errors bars indicate 95% confidence intervals. Numbers in parantheses indicate sample sizes for each genotype class.*

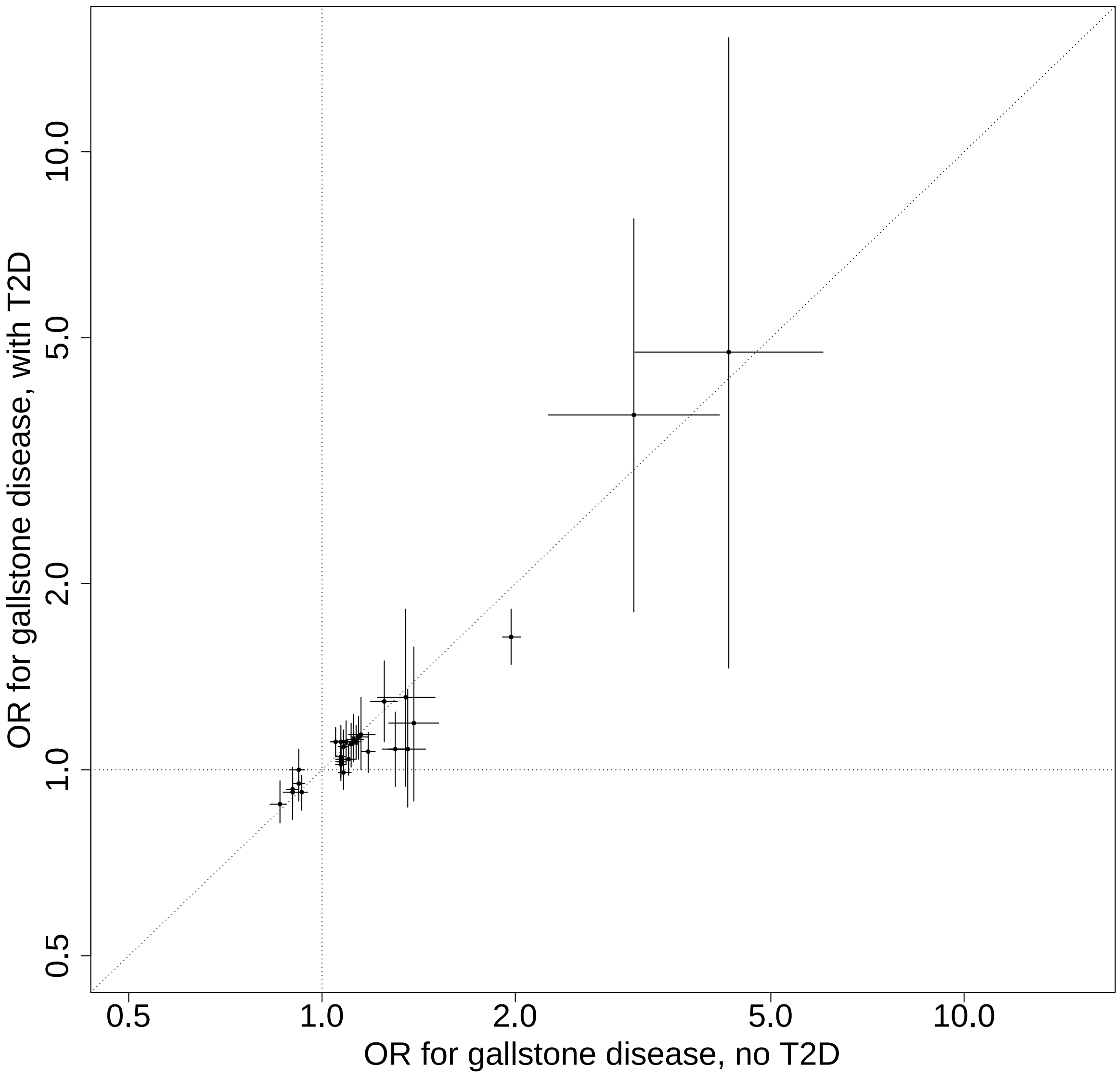

Supplementary Fig. 12. *A scatter plot showing gallstone disease odds ratios (OR) for the 32 variants found to associate with gallstone disease for type-2-diabetics (T2D) (x-axis) vs. non-diabetics (y-axis). There are no significant differences in gallstone disease OR between T2D cases and controls.*

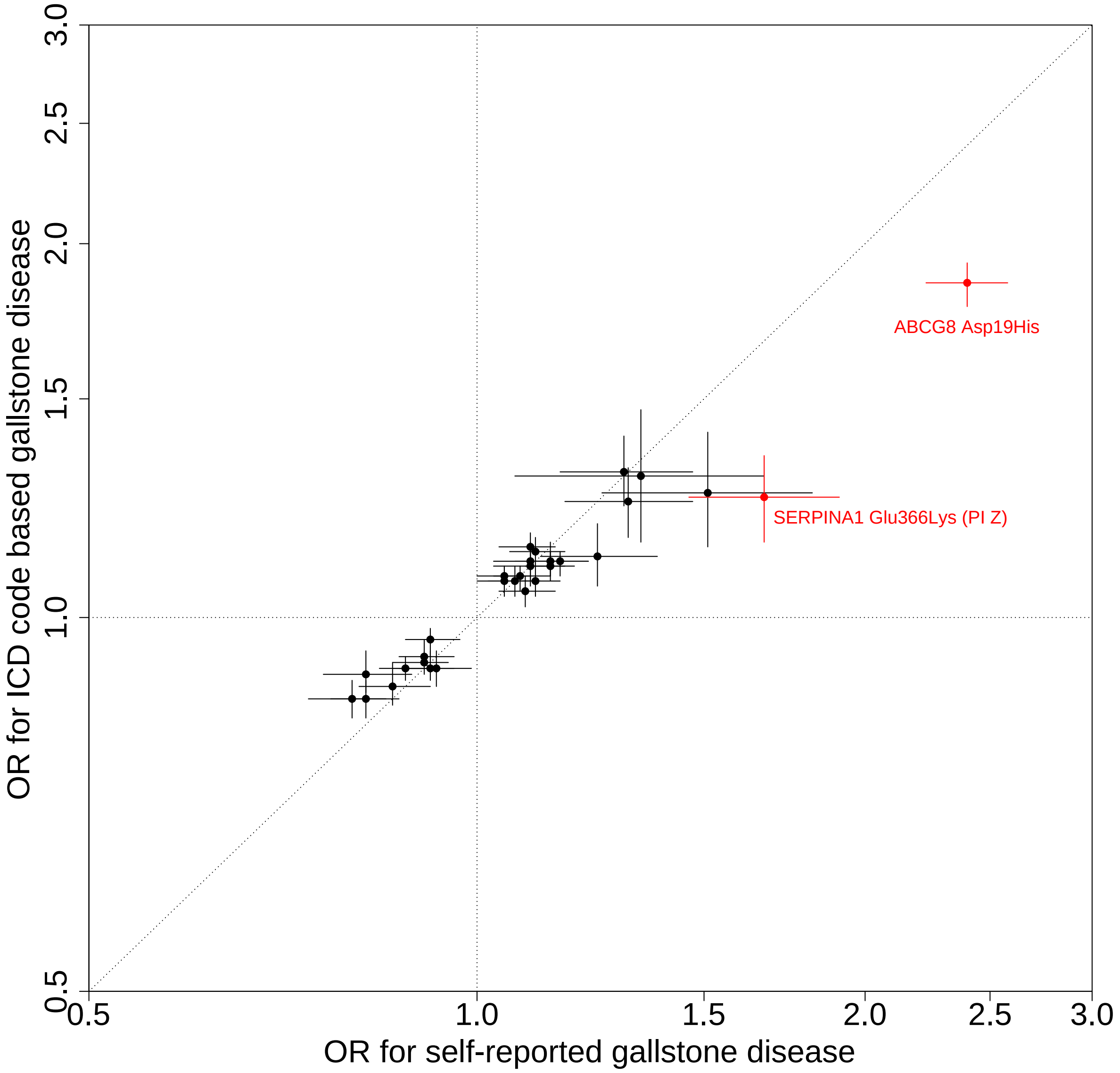

Supplementary Fig. 13. *A scatter plot showing showing gallstone disease odds ratios (OR) in the UK data for self-reported (x-axis) vs ICD code based (y-axis) gallstone disease cases.*
