# Supplementary material for: Genome-wide association meta-analysis yields 20 loci associated with gallstone disease
Source: Nat Commun. 2018 Nov 30;9:5101. doi: 10.1038/s41467-018-07460-y (PMC6269469; doi:10.1038/s41467-018-07460-y)
Supplement: Supplementary file 3 — Description of Additional Supplementary Files [file 41467_2018_7460_MOESM3_ESM.pdf]

Supplementary Data 1. Variants representing gallstone disease signals in the present meta-analysis of Icelandic and UK Biobank data. For Iceland (N-cases = 8,757 and N-controls = 346,688), UK BioBank (N-cases = 18,417 and N-controls = 390,150), in total N-cases= 27,174 and N-controls = 736,838). A chi-square test was used to test for association, effect is always shown for the minor allele. Significance levels and effects are shown separately for the Icelandic and UK BioBank datasets and for the combined analysis. MAF: Minor allele frequency, min: Minor allele, maj: Major allele, OR: Odds ratio, CI: Confidence interval, Phet: heterogeneity P-value.

Supplementary Data 2. Variants associating with gallstone disease in the present meta-analysis and their assocation with acute pancreatitis, cholecystitis, cholestasis of pregnacy and cirrhosis and fibrosis of liver, and gallbladder cancer. Effect is shown for the minor allele. MAF: Minor allele frequency. Amin/Amaj: Minor allele/Major allele. OR: odds ratio. Significant associations are highlighted in red.

Supplementary Data 3. Variants associating with gallstone disease in the present meta-analysis and their assocation with liver and lipid biomarkers. Effect is shown for the minor allele. MAF: Minor allele frequency. Amin/Amaj: Minor allele/Major allele. Effect: effect in standard deviations. ALT: alanine aminotransferase, AST: aspartate aminotransferase , GGT: gamma glutamyltransferase, ALP: alkaline phosphatase, TC: Total cholesterol, HDL: high-density lipoprotein, LDL: low-density lipoprotein. Significant associations are highlighted in red.

Supplementary Data 4. Supplementary Data 4. Sibling recurrence risk ratios (λS[i]) and population attributable fractions (PAF) for the risk alleles of the identified signals associated with gallstone disease in the present meta-analysis. We estimated the risk ratio among siblings (λS) of icelandic patients with gallstone disease (N = 2,628) by cross-matching with a genealogy database that covers the entire Icelandic nation. The risk ratio among siblings (λS) was estimated at 1.81 [1.71,1.91](P < 0.0001) using an approach previously described by Edvardsson et al. (Scand J Urol Nephrol, 2009, PMID:19921989). This allows us to calculate the proportion of sibling recurrence risk of gallstone disease explained by the signals identified in the present study, log(λS[i])/log(λS) (Witte et al, Nat Rev Genet, 2014 Nov, PMID:25223781).

Supplementary Data 5. Hypertriglyceridemia association in the Icelandic population of gallstone associated variants from the current study. An individual is defined as hypertriglyceridemic if at least two serum triglyceride measurements are over 5 mmol/L. P = P-value, OR = odds ratio, Effect: Effect in standard deviations.

Supplementary Data 6. Associations from the EBI GWAS catalog (e91, release 2018-01-28) for all the gallstone associated variants and their close correlates (R2 > 0.8) reported in the current study.

Supplementary Data 7. Non-coding variants in low LD class (n≤3) analysed with respect to enhancer associated characteristics (histone modifications, Dnase hypersensitivity sites and enhancer RNAs) in tissues relevant to the gallstone phenotype. The predicted gene targets for enhancers in relevant tissues are listed along with DNA binding proteins (ChIP-seq signals) intersecting with motifs disrupted by the association variants.

Supplementary Data 8. Inclusion criteria for gene enrichment analysis using Toppgene.

Supplementary Data 9. Associations reported to associate with Total cholesterol (TC), high-density lipoprotein (HDL) or low-density lipoprotein (LDL) and their association with gallstone disease in the combined analysis of Icelandic and UK data. The most significant associations in a megabase bin around a reported variant for TC, DHL or LDL are shown.

Supplementary Data 10. Gallstone association of variants in genes linked to maturity onset diabetes of the young (MODY). Comb_Pval = combined P-value for Iceland and UK, OR = combined odds ratio for Iceland and UK, omim_id = OMIM disease id, clinvar_pathogenicity = pathogenicity status of variant in the clinvar database, clinvar_disease = disease linked to variant in clinvar database.
